# How the Visual Cortex Handles Stimulus Noise: Insights from Amblyopia

**DOI:** 10.1371/journal.pone.0066583

**Published:** 2013-06-20

**Authors:** Éva M. Bankó, Judit Körtvélyes, Béla Weiss, Zoltán Vidnyánszky

**Affiliations:** 1 Faculty of Information Technology, Pázmány Péter Catholic University, Budapest, Hungary; 2 Department of Ophthalmology, Semmelweis University, Budapest, Hungary; 3 MR Research Center, Szentágothai J. Knowledge Center - Semmelweis University, Budapest, Hungary; 4 Department of Cognitive Science, Budapest University of Technology and Economics, Budapest, Hungary; University of Montreal, Canada

## Abstract

Adding noise to a visual image makes object recognition more effortful and has a widespread effect on human electrophysiological responses. However, visual cortical processes directly involved in handling the stimulus noise have yet to be identified and dissociated from the modulation of the neural responses due to the deteriorated structural information and increased stimulus uncertainty in the case of noisy images. Here we show that the impairment of face gender categorization performance in the case of noisy images in amblyopic patients correlates with amblyopic deficits measured in the noise-induced modulation of the P1/P2 components of single-trial event-related potentials (ERP). On the other hand, the N170 ERP component is similarly affected by the presence of noise in the two eyes and its modulation does not predict the behavioral deficit. These results have revealed that the efficient processing of noisy images depends on the engagement of additional processing resources both at the early, feature-specific as well as later, object-level stages of visual cortical processing reflected in the P1 and P2 ERP components, respectively. Our findings also suggest that noise-induced modulation of the N170 component might reflect diminished face-selective neuronal responses to face images with deteriorated structural information.

## Introduction

Human visual object recognition is fast and efficient when viewing conditions are good [1]–[3]. However, under deteriorated, suboptimal viewing conditions, which is often the case in natural circumstances, the visual system must recruit additional processing resources to handle the stimulus noise, thus object recognition becomes slower and more effortful [4]–[6]. Despite the numerous studies using noisy visual images, it is still unclear, which neural processes constitute the mechanism that is actively engaged by the visual system to enable or support successful recognition of objects when the visual input is noisy. These sensory processes that cope with stimulus noise are rather difficult to dissociate from other incidental processes also invoked by the noisy input e.g. to deal with increased stimulus uncertainty, task difficulty or decreased task-relevant information content, as they are inherently involved due to the nature of the stimulus thus, inseparable in studies on healthy subjects.

Indeed, it is well known, that altering the phase spectrum of face images, which contains most of the information about facial attributes [7] has a strong effect on the human visual cortical ERP responses [8]–[11]. In general, phase noise leads to a decreased N170 component, reflecting early structural face processing (for a review see [12] as well as to increased P1/P2 components, the latter of which might be associated with re-entrant higher level object processing mechanisms according to previous results [13]–[16]. Recently, we have shown [11] that these strong noise-induced response modulations cannot be accounted for by the changes in overall task difficulty as a result of adding phase noise to the stimuli, but instead, reflect the altered sensory processing of these images. However, the extent to which these noise-induced modulations reflect neural processes that are recruited to handle noisy images contributing to successful recognition or represent changes in feature specific neural responses owing to increased stimulus uncertainty is still unknown. Based on these findings we hypothesize that the noise-induced decrease of the N170 component might signal diminished responses from neurons coding the structural face information as a result of deteriorated face content, whereas the increase of the P1/P2 component might reflect the engagement of additional re-entrant visual cortical shape processing mechanisms in response to the inefficient structural information extraction.

To provide experimental support for this hypothesis we investigated the effect of phase noise on the ERP responses to face images in amblyopic patients. Previous behavioral and fMRI studies showed that in addition to the impaired low-level visual processing, amblyopia also involves higher-order, object-level processing deficits [17]–[20], which might result from sparse sampling at the level of the early visual cortex, spatial scrambling or increased positional uncertainty [20]–[22].

Based on this we predicted that neural processes engaged to handle the deteriorated shape information in the case of phase randomized face images are specifically impaired in amblyopia and will be identifiable as components with reduced noise-induced modulation in the amblyopic compared to the fellow eye. On the other hand, no interocular difference in noise-induced modulation of the N170 component is expected, if it primarily reflects diminished activity of face-responsive neurons coding structural face information, since face content of the images is equally deteriorated in both eyes as a result of decreasing the phase coherence by a fixed amount. In accordance with our hypothesis, our results revealed amblyopic deficits in face gender categorization in the amblyopic eye accompanied by a reduction in the noise-induced modulation of the P1 and P2 component of the ERP responses. On the other hand, the magnitude of the noise-induced modulation of the N170 component was similar in the two eyes.

## Materials and Methods

### Subjects

Nineteen amblyopic patients (mean±sd age: 30±8 years) gave their informed and written consent to participate in the study, which was approved by the ethics committee of Semmelweis University. However, one of them had to be excluded due to his poor performance on the task with both eyes, which left eighteen patients in total. All subjects were examined by an ophthalmologist and fitted with optimal correction. Table 1 details their medical parameters.

**Table 1:** Clinical details of amblyopic subjects.

		Refraction		Visual Acuity (VA)			
Subject	Age/Gender	RE	LE	RE	LE	Interocular VA (logMAR)	Squint
A1	32/F	−0.5	+0.5/+1.75 129°	20/12.5	20/80	0.8	ø
A2	25/F	−0.25/−0.5 135°	+3.75/+2.25 155°	20/16	20/80	0.7	ø
A3	20/F	+1.75/+1.25 101°	−1.0/+0.75 82°	20/80	20/20	0.6	ø
A4	36/M	plano	+2.5	20/12.5	20/63	0.7	ø
A5	24/M	−0.25/−1.75 97°	−3.0/−0.75 73°	20/80	20/16	0.7	ø
S1	38/F	+1.5/+1.75 91°	+2.5/+1.0 84°	20/20	20/40	0.3	ø
S2	34/F	+0.25/−0.25 12°	plano/−0.75 178°	20/63	20/12.5	0.7	D14Δ, N10Δ ET
S3	34/F	+1.25/−1.5 53°	+0.25/+0.25 62°	20/100	20/20	0.7	N = D10Δ ET
S4	29/F	−0.5	plano/−0.5 132°	20/16	20/40	0.4	ø
S5	22/M	−3.75/+3.5 159°	−2.25/+2.0 130°	20/20	20/32	0.2	ø
S6	22/M	+0.25	−0.25/−0.5 58°	20/80	20/10	0.9	D12Δ, N8Δ XT
S7	39/M	+1.25/−1.25 11°	+0.5/+1.5 95°	20/12.5	20/25	0.3	D8Δ, N8Δ ET
S8	23/M	+1.5/+1.25 100°	+2.75/+0.5 63°	20/40	20/12.5	0.5	D = N40Δ XT
S9	25/F	−4.25/−0.5 16°	−4.5/−0.75 176°	20/20	20/32	0.2	D = N25Δ XT
SA1	40/F	+1.75	+3.5	20/12.5	20/50	0.6	ø
SA2	46/F	−1.5/−1.0 140°	+0.25/−1.75 19°	20/20	20/125	0.8	D18Δ, N25Δ ET
SA3	22/M	+1.5	+3.0/+0.5 75°	20/10	20/63	0.8	D4Δ, N6Δ ET
SA4	24/M	+2.25/+1.0 177°	+3.75/+1.75 117°	20/16	20/32	0.3	D25Δ, N20Δ XT

A: anisometropic, S: strabismic, SA: strabismic & anisometropic, RE: right eye, LE: left eye, VA: visual acuity, D: distant, N: near, ET: esotropia, XT: exotropia. Patients listed as strabismics and having no squint angle, have been operated on after developing amblyopia.

### Visual Stimuli and Procedures

**Figure 1:**
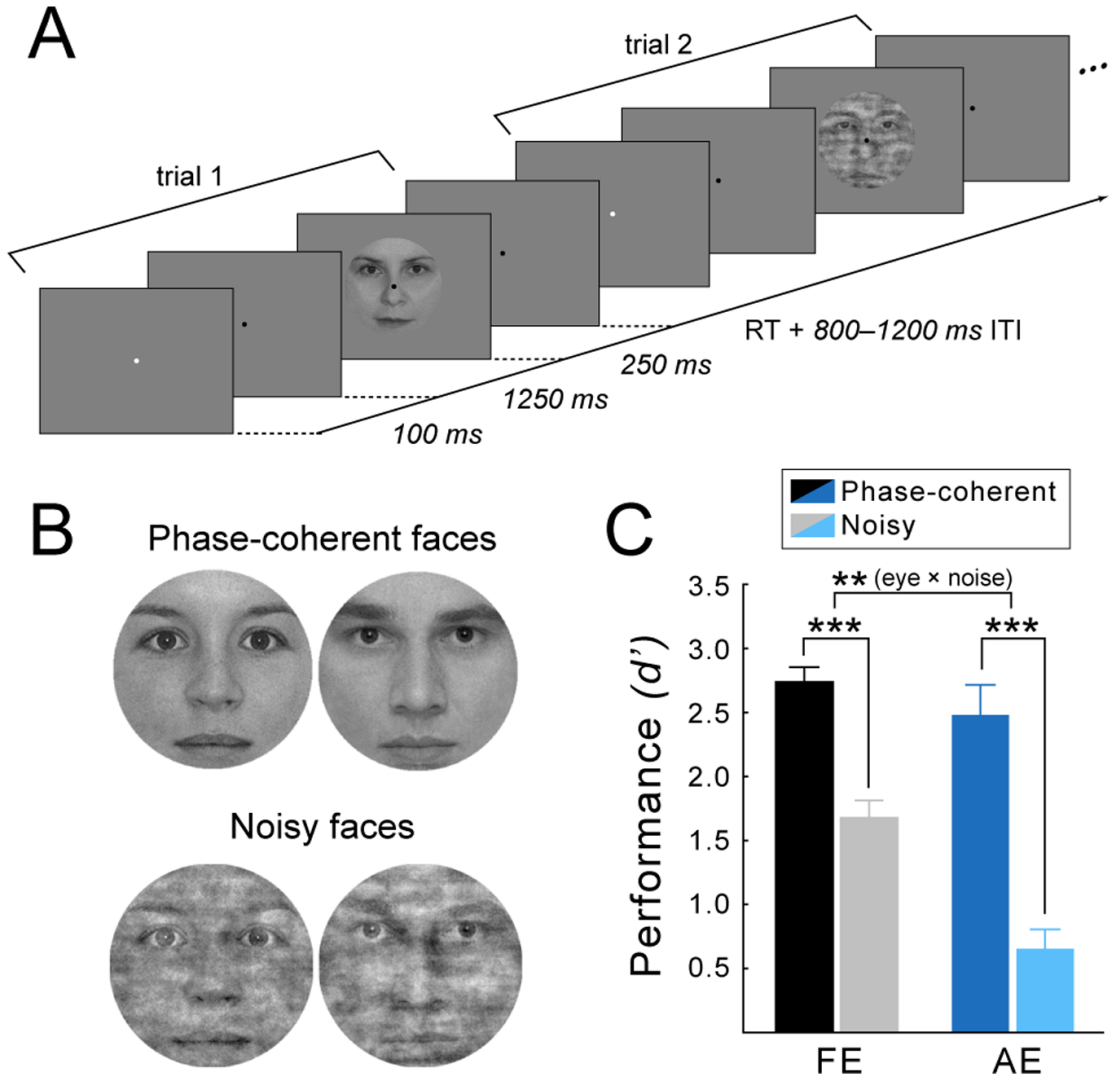
Stimuli, experimental protocol and behavioral results. (A) Experimental protocol, which shows the general stimulus sequence (two trials). (B) Exemplar gender pair for the phase-coherent and the 50% phase noise stimulus condition. The subjects of the photographs have given written informed consent, as outlined in the PLOS consent form, to publication of their photograph. (C) Phase noise impaired accuracy in both eyes, but the impairment was significantly greater in the amblyopic eye. FE: fellow eye, AE: amblyopic eye. Error bars indicate ±SEM (N = 18, ** *p*<0.01, *** *p*<0.001).

Participants performed a two-alternative forced choice gender categorization task with morphed female/male face images with 100% and 50% phase coherence (phase-coherent face condition: PC and noisy face condition: N, respectively) subtending 2 degrees (approx. the size of the fovea). Four female and four male images were chosen for the experiment. Images were taken from our face database which was obtained with written informed consent to publication of their photographs. Warping was done in WinMorph 3.01, while phase coherence was manipulated using custom made scripts based on the weighted mean phase technique [11], [23] (Figure 1B shows an exemplar face pair for both stimulus conditions; a detailed description of image processing can be found in Bankó et al. [11], [24]). Based on pilot sensitivity measures the gender difference (i.e. morph level) between female and male stimuli was adjusted separately for the amblyopic and fellow eye in each observer (typically 25/75% and 5/95% gender content for the fellow and amblyopic eye, respectively; for individual morph levels see Fig. S1) to achieve similar gender categorization performance (80–90% accuracy) for the two eyes in the phase-coherent face condition. The phase coherence of these morphed face images was then decreased to 50% for the noisy face condition. Adding equal amount of phase noise to the performance equated morphed images allowed us to compare the noise induced performance decrement across the fellow and the amblyopic eye without the confounding initial gender categorization performance difference between eyes.

Each trial started with a cue, a brief change (100 ms) in the color of the fixation dot followed by the face stimulus for 250 ms with a fixed SOA of 1350 ms on 80% of the total trials and 2350 ms on 20% of the trials. Subjects were instructed to pay attention following the cue and were explicitly told about the 1350 ms SOA but were not informed about the extra 1 s delay in 20% of the trials These latter trials were used for calculating the interocular oscillatory baseline differences within the timeframe of the cue and the expected stimulus, since they did not contain a stimulus evoked response at the time of the expected stimulus onset (for more explanation and results see [24]). A response window of 2 s was given, which terminated when the subjects responded. Trials were separated by a random ITI of 800–1200 ms (Fig. 1A). A fixation dot was present throughout the entire block; stimuli were presented centrally on a uniform gray background. The noisy (N) and phase-coherent (PC) conditions were presented with equal probability within a block in random order. Viewing was monocular, alternating between blocks, while the other eye was patched. Each participant completed four runs for each eye yielding 192 trials altogether for each stimulus type per eye and altogether 80 trials per eye where the face images where delayed. Stimulus presentation was controlled by MATLAB 7.1. (The MathWorks Inc., Natick, MA) using the Cogent 2000 toolbox (http://www.vislab.ucl.ac.uk/cogent_2000.php) and were presented on a 26″ LG LCD monitor at a refresh rate of 60 Hz and were viewed from 56 cm.

### Electrophysiological Acquisition and Processing

EEG data was acquired using a BrainAmp MR (Brainproducts GmbH., Munich, Germany) amplifier from 60 Ag/AgCl scalp electrodes placed according to the extended 10–20 international electrode system, mounted on an EasyCap (Easycap GmbH, Herrsching-Breitbrunn, Germany) with four additional periocular electrodes placed at the outer canthi of the eyes and above and below the right eye for the purpose of recording the electrooculogram. All channels were referenced to joint earlobes online; the ground was placed on the nasion. All input impedance was kept below 5 kΩ. Data were sampled at 1000 Hz with an analog bandpass of.016–250 Hz and was re-referenced offline using a Laplacian transform on spherical spline interpolated data to generate scalp current density (SCD) waveforms. The SCD data is reference independent and displays reduced volume conduction eliminating raw EEG contamination from saccadic potentials [25], [26]. Moreover its peaks and troughs are sharper and larger than those of the original scalp potential [27], which makes it better suited for single-trial peak detection compared to raw surface potentials [28]. Data were band-pass filtered from.1–30 Hz (using digital.1 Hz 12 dB/octave Butterworth Zero Phase high-pass filter, 30 Hz 24 dB/octave low-pass filter, and 50 Hz notch filter), segmented, artifact rejected and baseline corrected in a 200 ms pre-stimulus window directly preceding the presentation of the stimulus. 1000-ms long epochs (−200–800 ms relative to stimulus) were used for creating the trial-averaged event-related potentials and for single trial peak analysis. Data processing was done using BrainVision Analyzer (Brainproducts GmbH., Munich, Germany).

### Statistical Analysis

**Figure 2:**
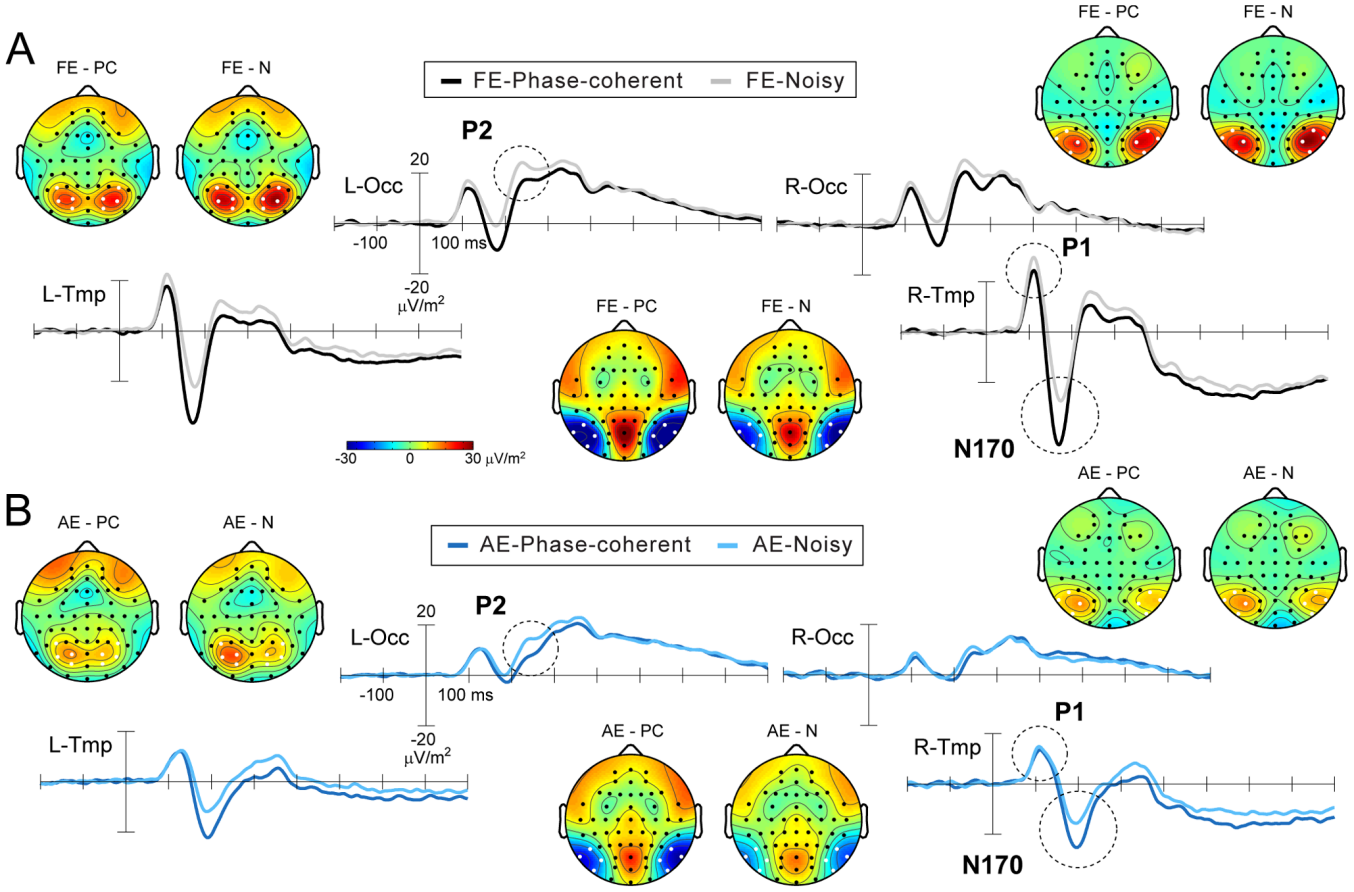
Grand-average event-related potentials (ERP) to faces from (A) the fellow and (B) the amblyopic eye. Trial-averaged waveforms are displayed as clusters averaged from electrodes marked with white dots on the topographical maps. Peak topographic maps are displayed at the time of their respective maxima/minima. P1 and N170 were analyzed over bilateral temporal clusters (L/R-Tmp) while P2 was analyzed over bilateral occipital clusters (L/R-Occ). Note, that cartoon heads are plotted with unrealistic head radius for better electrode visibility. FE: fellow eye, AE: amblyopic eye, PC: phase-coherent, N: noisy.

Accuracy was assessed calculating d-prime. P1, N170 and P2 component peaks were detected and analyzed on electrodes clustering around the ones showing maximum deviation relative to baseline in the group average in the expected time period corresponding to the ERP peaks. The clusters coincided for P1 and N170 (PO7, PO9, P7, and P9, and PO8, PO10, P8, and P10 for left and right clusters, respectively), while for P2 different clusters were used (P5, PO3, PO7, and O1, and P6, PO4, PO8, and O2, for left and right clusters, respectively). The somewhat unusual choice of electrodes for component P1 (posterior-temporal instead of occipital) is due to the SCD transform slightly altering topographies shifting the maximum posterior-temporally (see Fig.2 for component topographies). Peaks were detected on each trial for each electrode as maximum and minimum activity for P1/P2 and N170, respectively in an 80 ms time window centered on the individual peak latency of the respective component measured on the averaged ERPs, which was determined on pooled electrodes from left and right clusters separately. The amplitude and corresponding time of the extremes were taken as the amplitude and latency of the component on a given trial. The trial was rejected if the detected extreme was located at the beginning or end of the time window. The single trial amplitude and latency values were pooled from the four electrodes on each side and the distribution of the values was characterized by calculating the median and the interquartile range (IQR), which is a measure of spread and is computed as the difference of the upper and lower quartile of the data, and thus describes the middle 50% of the data values. IQR was deemed to be a good choice since it is a robust measure, i.e. insensitive to outliers (unlike standard deviation) and does not assume symmetric distributions (as opposed to median absolute deviation).

The above measures were compared using repeated-measures ANOVAs with within-subject factors of eye (fellow: FE vs. amblyopic: AE), phase coherence (100%: PC vs. 50%: N) for the behavioral measure with additional within-subject factors side (L vs. R) for the electrophysiological measures, using Tukey HSD tests for post-hoc comparisons. Homogeneity of variances was tested using F-tests and in case this assumption was not met due to the higher variance of measurements from the AE, values were first rank transformed before being entered into the statistical test, which is noted by rANOVA (rank ANOVA) when detailing statistical results.

We assessed the relationship between the noise-induced changes in the component distribution medians of the two eyes using Spearman rank correlation, which is relatively insensitive to the contribution of outliers. We also assessed the relationship between the performance decrease and the median increase of each component. The noise-modulation index was expressed as percent increase relative to the phase-coherent condition for the P1/P2 medians 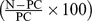 and percent decrease for N170 medians and performance 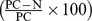. The ERP of one subject was such that the P2 component barely reached positivity, thus rendering the index in her case senseless. Therefore, we excluded her from the correlation analyses that involved the P2 component.

### Analysis of Eye-tracking Data

We tracked the gaze direction of all subjects using the iViewX Hi-Speed tracking column (SMI GmbH, Teltow, Germany) while they performed the EEG experiment. However, we were able to record useable eye movement data only for ten patients due to the strong reflection of glasses that many were wearing. Eye-gaze direction was assessed using a summary statistic approach. Trials were binned based on the viewing eye and stimulus phase coherence, while mean eye position (x and y values) was calculated for periods when the face stimulus was present on each trial. From each of the four eye-gaze direction dataset, spatial maps of eye-gaze density were constructed. The root mean squares (RMS) of the density values for these maps were computed [29], as a measure of fixation stability, higher RMS values meaning less stable fixation. Data was analyzed with a two-way repeated-measures ANOVA with eye and phase coherence as within subject factors.

## Results

### The Effect of Noise on Face Gender Categorization

Adding noise to the face images resulted in a significant drop in face gender categorization performance as compared to the performance with intact, phase-coherent faces in both eyes (Fig1C.; rANOVA, main effect of noise: F_(1,17)_ = 114.22, *p*<.0001). More importantly, however, the noise-induced performance decrement was more pronounced for the amblyopic than for the fellow eye: accuracy did not differ significantly between eyes in the case of phase-coherent faces while there was a marked performance difference between eyes in the case of noisy faces (rANOVA, eye × noise interaction: F_(1,17)_ = 14.74, *p* = 0.0013, post-hoc PC_FE_ vs. PC_AE_ *p* = 0.096 while N_FE_ vs. N_AE_ *p* = 0.0002). Nevertheless, categorization of noisy faces seen with the amblyopic eye was still significantly above chance (t-test against reference mean of 0.0: t_(17)_ = 4.35, *p* = 0.0004). Our results thus revealed that noise impairs face gender categorization performance in the amblyopic eye to a greater extent compared to the fellow eye, since categorization performance of the original, phase-coherent face stimuli was adjusted to be equal in the two eyes (see ‘Visual stimuli and procedures’ section). These behavioral findings suggest that the neural mechanisms involved in the processing of noisy face stimuli with deteriorated contour information might be impaired in amblyopia.

### Noise-induced Changes in Component Amplitude- and Latency Distributions Derived from Single-trial Analysis

Even though the magnitude of the ERP component amplitudes derived from trial-averaged ERPs is affected by the latency jitter of the ERP components across trials [30], most ERP research utilizes the robustness of trial-averaged event-related potentials, as there is no reason to assume that this latency jitter is affected differently across conditions. However, there are cases where increased latency jitter might be a serious concern such as autism [28] and even more so amblyopia [24]. In fact, the possibility that impaired temporal structure of neural responses might contribute to the severe amblyopic amplitude decrease found in the ERP results [31]–[34] is supported by previous findings showing that in strabismic cats neuronal response latencies could be more variable in visual cortical neurons driven by the amblyopic eye [35]–[37] as well as by the human electrophysiological results revealing increased latency jitter of the ERP components across trials in the amblyopic as compared to the fellow eye [24]. To circumvent this possible confound, we performed a single-trial ERP analysis, detecting peaks on each trial, which enabled us to investigate the effect of noise sensitivity in the trial-by-trial amplitude and latency of the ERP components. Findings pertaining to the overall differences between the fellow and amblyopic eye are not the focus of the present paper and are not discussed here. For those results we kindly refer the reader to Bankó et al. [24].

#### P1 and P2 component distributions

**Figure 3:**
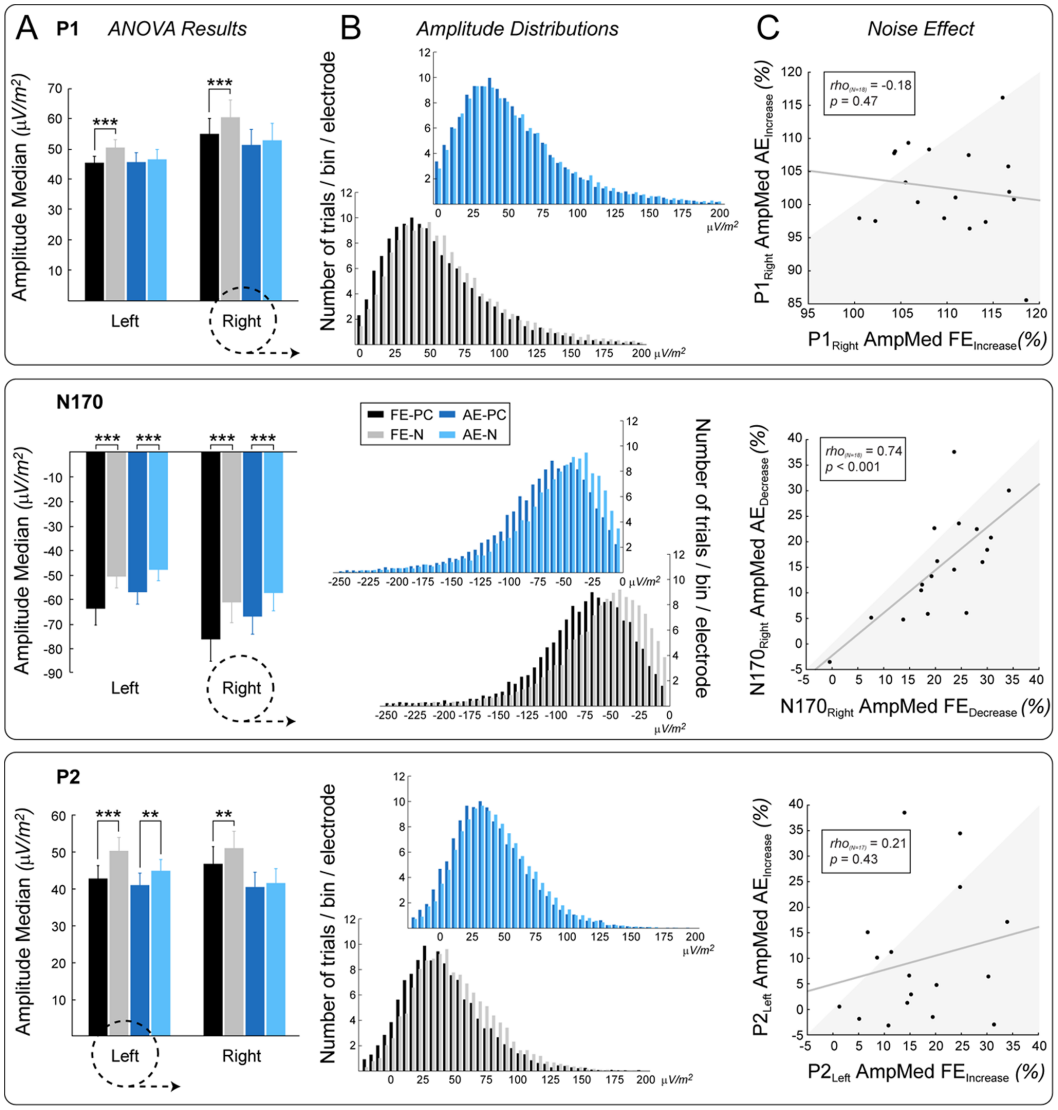
Results of the single-trial analysis. (A) Statistical analysis of the amplitude medians of components P1 (top panel), N170 (middle panel) and P2 (bottom panel). (B) Amplitude distributions collected from right side cluster electrodes in the case of P1 and N170 and left side cluster electrodes in the case of P2, as indicated by dashed circles in panel A. (C) Spearman correlations between the magnitude of the noise-induced amplitude median increase/decrease observed in the fellow and amblyopic eye for each component over the hemisphere indicated by dashed circles in panel A. The shaded area denotes bigger noise-induced change in the fellow eye relative to the amblyopic eye. FE: fellow eye, AE: amblyopic eye, PC: phase-coherent, N: noisy. Error bars indicate ±SEM (N = 18 unless indicated otherwise; **p<.01, ***p<.001).

In accordance with previous results [8], [9], [11], the presence of noise strongly affected visual cortical processing of face stimuli, reflected in the P1-N170-P2 ERP complex (Fig.3; rANOVA, main effect of noise on amplitude medians: F_(1,17)_ = 30.50, *p*<0.0001; F_(1,17)_ = 70.13, *p*<0.0001 for components P1 and N170 respectively, and ANOVA main effect of noise: F_(1,17)_ = 58.51 *p*<0.0001 for components P1, N170 and P2, respectively). More importantly, however, the modulation of the P1 and P2 amplitudes due to the addition of phase noise differed between the amblyopic and fellow eye: there was a reduction in the noise-induced modulation of the P1 and P2 component amplitudes in the amblyopic eye as compared to the fellow eye. In the noisy condition, P1 amplitude distributions were shifted towards larger values – as indicated by an increase in medians – only under normal but not under amblyopic viewing conditions (rANOVA, eye × noise interaction: F_(1,17)_ = 17.83, *p* = 0.0006, post-hoc PC_FE_ vs. N_FE_ *p* = 0.0002 while PC_AE_ vs. N_AE_ *p* = 0.81). On the other hand, P2 amplitude distribution medians increased for both eyes when viewing noisy faces compared to phase-coherent faces. Albeit present, the shift was significantly smaller in the amblyopic compared with the fellow eye (ANOVA, eye × noise interaction: F_(1,17)_ = 15.24, *p* = 0.0011, post-hoc PC_FE_ vs. N_FE_ *p* = 0.0002 and PC_AE_ vs. N_AE_ *p* = 0.0037). Moreover, this effect was significantly larger over the left hemisphere for both eyes (ANOVA, noise × side interaction: F_(1,17)_ = 6.33, *p* = 0.022, post-hoc PC_Left_ vs. N_Left_ *p* = 0.0002 and PC_Right_ vs. N_Right_ *p* = 0.025), which suggests that the left hemisphere is more effectively engaged in the additional processing levied in the noisy condition. For the amblyopic eye, the amplitude increase was in fact only significant over the left hemisphere (PC_AE_ vs. N_AE_ planned comparison: F_(1,17)_ = 12.04, *p* = 0.0029 and F_(1,17)_ = 1.86, *p* = 0.19 for left and right hemispheres, respectively).

**Figure 4:**
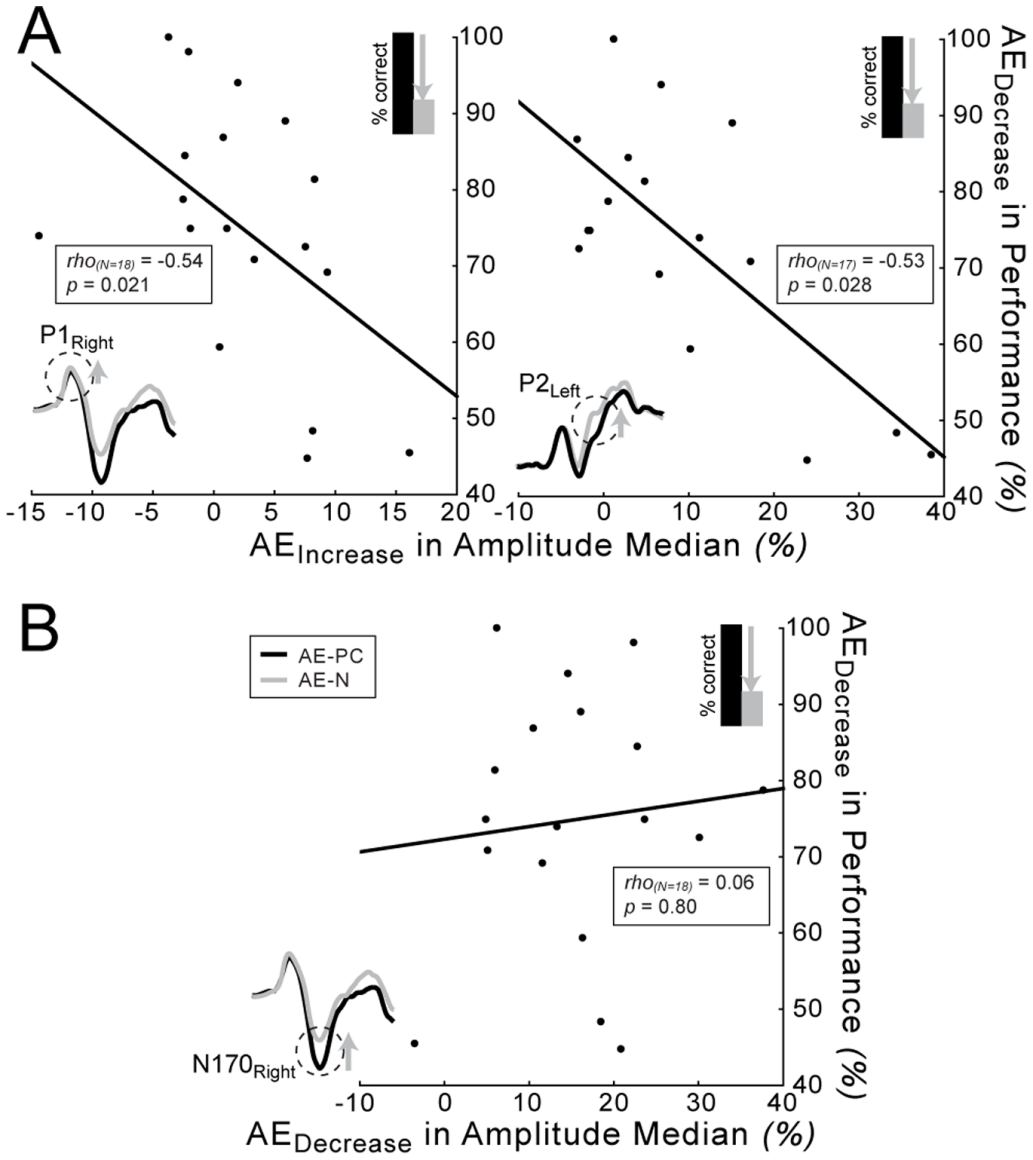
Noise effect on behavior and amplitude medians. (A) Spearman correlation between the noise-induced increase in P1 and P2 amplitude medians over the right and left hemisphere, respectively and the noise-induced decrease in performance of the amblyopic eye. Negative correlation indicates that the larger the P1/P2 amplitude increase in a subject, the smaller the performance decrement. (B) Same correlation as in panel A for amplitude median decrease of the N170 component. AE: amblyopic eye, PC: phase-coherent, N: noisy.

Importantly, this amblyopic noise effect on the P1 and P2 amplitude medians measured over the right and left hemisphere, respectively – as expressed by percent amplitude increase in the noisy relative to the phase-coherent condition – negatively correlated with the noise-induced percent decrease in gender categorization performance in the amblyopic eye: the larger the increase in P1/P2 amplitude medians, the smaller the detrimental effect of noise on performance (Fig. 4A; Spearman rank correlation rho_(N = 18)_ = −0.54, *p* = 0.022 and rho_(N = 17)_ = −0.53, *p* = 0.028 for P1 over the right and P2 over the left hemisphere, respectively). The noise-induced increase in amplitude medians of these components also tended to correlate positively with each other, which however, did not reach significance (rho_(N = 17)_ = 0.43, *p* = 0.083). On the other hand, we found no such correlation between the effect of noise on behavior and neural responses (P1/P2) in the case of the fellow eye (all |rho|≤0.26, *p*≥0.29). The fact that we found correlation between the P1/P2 increase and performance decrement only in the amblyopic but not in the fellow eye suggests that P1/P2 components reflect a subset of the neural processes involved in the processing of noisy images, which are specifically damaged in amblyopia.

As opposed to the amplitude distributions, introducing phase noise affected the latency distributions of both components similarly across eyes (rANOVA/ANOVA eye × noise interaction: all F≤1.50 and *p*≥0.24), even though it did have an overall effect on the distributions. Noise induced a small latency shift and a slight but insignificant increase in the latency jitter of the P1 component (rANOVA main effect of noise: F_(1,17)_ = 7.0, *p* = 0.017 and F_(1,17)_ = 3.26, *p* = 0.09 on latency medians and latency jitter, respectively), while it had no effect on the latency but increased the latency jitter of the P2 component (ANOVA main effect of noise: F_(1,17)_ = 1.80, *p* = 0.20 and F_(1,17)_ = 10.4, *p* = 0.005 on latency medians and latency jitter, respectively).

#### N170 component distributions

Importantly, no interocular differences were found in the noise effects on the N170 amplitudes: the noise-induced amplitude decrease of the N170 was evident under both viewing conditions (rANOVA main effect of noise: F_(1,17)_ = 70.13, *p*<0.0001; eye × noise interaction: F_(1,17)_ = 2.96, *p* = 0.10). The lack of eye × noise interaction is complemented by the strong correlation found between the noise effects (percent change) observed in the fellow and amblyopic eyes in the case of the N170 component (Spearman rank correlations: rho_(N = 18)_ = 0.57, *p* = 0.013 and rho_(N = 18)_ = 0.74, *p*<0.001 for left and right hemisphere, respectively; Fig. 3). However, there was no such correlation between the noise effect in the amblyopic and fellow eyes for components P1 and P2 (all |rho_(N = 18)_|≤0.26 and *p*≥0.29 and |rho_(N = 17)_|≤0.21 and *p*≥0.43 for P1 and P2, respectively; Fig. 3). There was also no connection between the effect of noise on the N170 amplitude medians and the behavioral deficit for either eye (Fig. 4B; all |rho_(N = 18)_|≤0.20, *p*≥0.42). Phase noise led to a similar increase in N170 latency medians and jitters in the two eyes (rANOVA/ANOVA eye × noise interaction: all F≤1.43 and *p*≥0.25; rANOVA main effect of noise on latency median: F_(1,17)_ = 22.1, *p* = 0.0002; ANOVA main effect of noise on latency jitter: F_(1,17)_ = 17.34, *p* = 0.0006).

Taken together, our results revealed that the noise-induced modulation observed for stimuli presented to the amblyopic and fellow eye differs only in the case of the P1 and P2 but not in the case of the N170 component.

### Results of the Eye-tracking Analysis

The results revealed that in agreement with previous findings [24], [38]–[40] fixations were more stable in the case of the fellow eye as compared to the amblyopic eye (main effect of eye: F_(1,9)_ = 9.26, *p* = 0.014). However, this was not a serious concern, since the difference in fixation stability was found to correlate only with component latency but not with component amplitude [24]. On the other hand, fixation was not affected by adding noise to the face images, neither was there any interaction between the two factors (main effect of noise: F_(1,9)_ = 1.92, *p* = 0.20; eye × noise interaction: F_(1,9)_ = 0.014, *p* = 0.91).

## Discussion

The results revealed strong amblyopic deficits in visual cortical processing of phase randomized face images. Adding phase noise to the stimuli resulted in a larger drop of face gender categorization performance and a smaller increase of P1 and P2 amplitudes when viewing with the amblyopic eye as compared to the fellow eye. Furthermore, the behavioral effect of noise negatively correlated with the strength of noise-induced modulation of both components, suggesting that the inefficiency of an early, feature-specific stage of visual cortical processing reflected in the P1 component and a later stage of object processing reflected in the P2 component both contribute to the impaired processing of noisy images in amblyopia. On the other hand, the magnitude of noise-induced modulation of the N170 component was comparable in the two eyes and showed a strong interocular correlation. This implies that structural level processing of face stimuli, reflected in the N170 component (for a review see [12] is similarly affected by noise in the case of the amblyopic and fellow eye.

Since the P1 component in the case of face processing is driven primarily by the low-level visual cues but not by structured information associated with the percept of a face [41]–[43], these findings suggest that the noise-induced modulation of early, low-level visual cortical processes is also altered in amblyopia. This is further supported by the fact that the behavioral effects of noise correlated with the individual variations in P1 noise-modulation of the amblyopic eye measured over the right hemisphere, since early face processing involving the P1 and N170 component is right lateralized. This is in accordance with the known low-level visual processing deficits of amblyopia [31]–[34].

Furthermore, significant noise-induced amblyopic deficits were observed in the P2 component, which reflects the engagement of a later stage of object processing [13], [15], [44], [45] presumably involving re-entrant shape processing mechanisms in a retinotopically organized region of the lateral occipital cortex [11], [14]. It is also thought to be associated with grouping processes [16], [46]. Phases in an image carry location information, which in turn specifies object shape in terms of the spatial locations of features [47], [48] and this feature location information is crucial for categorizing objects [49]. Therefore, our results showing inefficient handling of the disruptive effect of phase noise in amblyopia are in agreement with the previous behavioral findings that identify undersampling, spatial scrambling, and increased positional uncertainty as key characteristics of the amblyopic vision [20]–[22]. Moreover, they provide the neurophysiologic background for the reduced ability to processes deteriorated shape information in the case of phase randomized face stimuli as well as the first neurophysiological evidence for impairement in visual cortical processing beyond the early stage of object recognition in amblyopes.

The deficit in noise-modulation measured on the P1/2 components also tended to correlate with each other, raising the question whether the latter is not a simple carry over effect of the first. Even though we cannot exclude the possibility that the deficit measured on P1 affects the deficit measured on P2, the following argue against a simple carry over effect: i) The noise-induced amplitude increment of the P1 component cannot be found for the amblyopic eye, while that of the P2 component reaches significance in the amblyopic eye (over the left hemisphere only); ii) In addition, the N170 component, separating the P1 and P2 components displays roughly the same noise effect in both eyes; iii) Moreover, according to knowledge accumulated about the possible processes the P2 component reflects, the amblyopic deficits reflected in this component could be a good candidate for the neurophysiologic background for the reduced ability to processes deteriorated shape information in the case of phase randomized face stimuli.

Interestingly, amblyopic deficits were found only in the noise-induced modulation of the amplitudes but not in that of the latencies of the ERP responses, which supports the idea that the amblyopic effects on the strength and on the timing of the visual cortical responses might reflect different neural dysfunctions [24].

Our findings have broader implications concerning the neural processes that are engaged by the visual system when facing deteriorated, noisy images. In previous research, adding noise to the stimuli was used extensively to study the degree of feature or object category selectivity of a specific neural population or visual cortical area [9], [42], [43], [50] as well as to manipulate stimulus uncertainty and overall task demands in order to reveal the neural processes underlying accumulation of sensory evidence and computation of decision variables [8], [51]–[54]. However, how the visual system handles stimulus noise has received much less attention and thus the nature of the visual cortical processes that are recruited when noisy, degraded visual images have to be categorized and discriminated is still unclear. Previous human neurophysiological studies have shown that noise has a strong effect on the early visual cortical responses, reflected in the EEG and MEG responses over the visual cortex, in a temporal interval ranging from 100–300 ms following stimulus onset [8], [9], [11], [55]. The most consistent finding is that adding noise to the stimulus leads to: i) reduced activity in the 130–200 interval after stimulus onset, corresponding to the N1/M1 component (N170 in case of faces) of the ERP/MEG responses and ii) increased activity in a later temporal interval (between 200–300 ms), corresponding to the P2 component.

The time window of the N170 component reflecting the structural level processing of visual objects [12] has been found to correspond to maximum noise sensitivity in healthy young adults [10], [56], that is the effect of noise-modulation appears to be largest around 150 ms, which is also in line with our results. However, the fact that the N170 was modulated by noise similarly between eyes and the highly significant interocular correlation of the noise effect suggest that the noise-induced changes in the structural processing of visual objects might primarily reflect the decrease in facial content of the images as a result of phase noise. Moreover, the noise-induced behavioral decrement correlates with the noise effect on the ERP amplitudes both before (P1) and after (P2) this time period, however, does not on the N170. Thus it seems unlikely that the N170 would correspond to the time point at which the additional sensory processes are maximally engaged. Rather, the observed maximum noise effect around 150 ms most likely represents a combination of active noise processing and a diminished activity of face-responsive neurons coding the structural face information, which happens concomitantly as a result of decreasing the phase coherence of stimuli.

Our results suggest that the noise-induced modulation of the P2 might reflect a critical component of visual cortical processes that is recruited to handle stimulus noise after core, structural processing of the visual object – reflected in the N170 component – has been completed. It is important to note, however that adding noise to the stimulus will not only increase the visual cortical processing demands but will also result in enhanced responses of the neural populations representing stimulus uncertainty [57]. As a matter of fact, in all previous studies the noise-induced modulation of the P2 component could be explained by either of these two factors. The results of the current study showing that fine discrimination of objects embedded in visual noise is accompanied by reduced noise effects on the P2– and also the P1– component in the amblyopic compared to the fellow eye appears to be at odds with the stimulus uncertainty account of the noise-induced modulation of the P2 component, as one would expect a similar or even more pronounced noise-modulation in the amblyopic eye, if the P2 component increment reflected enhanced responses of the neural populations representing stimulus uncertainty, since stimulus uncertainty was similarly increased in the two eyes by adding the same amount of noise to the stimuli. Furthermore, these results also exclude the possibility that noise-induced increase of P2 component is due to the enhanced overall task difficulty suggested earlier [8], as face gender categorization performance was lower – i.e. task difficulty was higher – when stimuli were presented to the amblyopic eye as compared to the fellow eye.

The results of the current study concern diminished noise-modulation of signal from the amblyopic eye, thus it is important to consider the signal-to-noise ratio (SNR) in the two eyes. VEP and ERP studies have consistently reported drastically reduced responses from the amblyopic eye in comparison to the fellow eye, which could indicate decreased SNR in the case of the amblyopic eye, which in turn could result in decreased noise-modulation. However, it is important to note that we have recently shown that diminished ERP amplitudes are largely due to an increase in trial-to-trial latency jitter in the amblyopic eye, thus they arise as an artifact of averaging. In fact, the true trial-to-trial response amplitudes in the amblyopic eye uncovered by single-trial peak detection revealed no or only a slight reduction in the case of the P1 and N170, respectively, compared to the fellow eye (for detailed results and statistics see [24]). This is also illustrated in the bar diagrams of peak amplitude medians in Fig.3A. Moreover, there were also no differences in oscillation power and phase distribution preceding the face stimuli [24]. Taken together, this strongly argues against a difference in SNR between the two eyes and so an SNR reduction account of the amblyopic effect on noise-modulation.

### Conclusions

The present results suggest that in case the visual images are noisy and/or deteriorated, core object processing, taking place within the first 200 ms of after stimulus onset, is strongly diminished due to the decreased structural information content of the images, which is reflected in the reduced amplitudes of N170 component. As a result, additional processing resources – presumably involving re-entrant mechanisms residing in the lateral occipital cortex – have to be actively engaged, which are manifested in the increased amplitudes of the P1 and P2 component, the impairment of which will lead to behavioral deficits in fine discrimination of objects embedded in noise as seen in amblyopic patients.

## Supporting Information

Figure S1 **Perceptual balancing of stimuli.** Individual mean morph levels of the fellow eye (FE) and the amblyopic eye (AE). Morph levels were adjusted to achieve similar gender categorization performance (80–90% accuracy) for the two eyes in the phase-coherent face condition. Gender content was typically 25/75% and 5/95% for the fellow and amblyopic eye, respectively, which is shown on the top panel. The subjects of the photographs have given written informed consent, as outlined in the PLOS consent form, to publication of their photograph.
(DOC)
