# Supplementary material for: How the Visual Cortex Handles Stimulus Noise: Insights from Amblyopia
Source: PLoS One. 2013 Jun 20;8(6):e66583. doi: 10.1371/journal.pone.0066583 (PMC3688592; doi:10.1371/journal.pone.0066583)
Supplement: Figure S1 — Perceptual balancing of stimuli. Individual mean morph levels of the fellow eye (FE) and the amblyopic eye (AE). Morph levels were adjusted to achieve similar gender categorization performance (80–90% accuracy) for the two eyes in the phase-coherent face condition. Gender content was typically 25/75% and 5/95% for the fellow and amblyopic eye, respectively, which is shown on the top panel. The subjects of the photographs have given written informed consent, as outlined in the PLOS consent form, to publication of their photograph. (DOC) [file pone.0066583.s001.doc]

Figure S1.


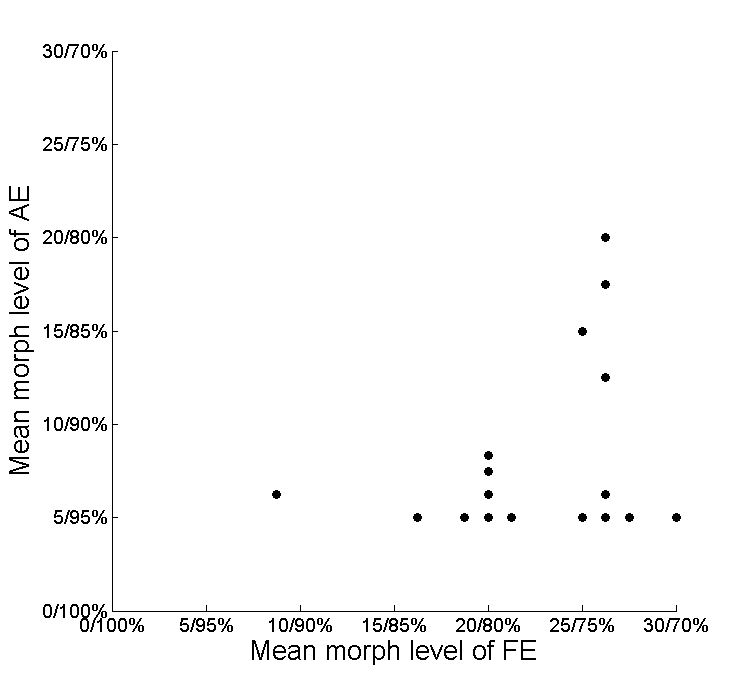


**Figure S1.** Perceptual balancing of stimuli. Individual mean morph levels of the fellow eye (FE) and the amblyopic eye (AE). Morph levels were adjusted to achieve similar gender categorization performance (80-90% accuracy) for the two eyes in the phase-coherent face condition. Gender content was typically 25/75% and 5/95% for the fellow and amblyopic eye, respectively, which is shown on the top panel. The subjects of the photographs have given written informed consent, as outlined in the PLOS consent form, to publication of their photograph.
